# Living Bacterial Sacrificial Porogens to Engineer Decellularized Porous Scaffolds

**DOI:** 10.1371/journal.pone.0019344

**Published:** 2011-04-28

**Authors:** Feng Xu, BanuPriya Sridharan, Naside Gozde Durmus, ShuQi Wang, Ahmet Sinan Yavuz, Umut Atakan Gurkan, Utkan Demirci

**Affiliations:** 1 Demirci Bio-Acoustic-MEMS in Medicine (BAMM) Laboratory, Department of Medicine, Center for Biomedical Engineering, Brigham and Women's Hospital, Harvard Medical School, Boston, Massachusetts, United States of America; 2 Division of Biology and Medicine, School of Engineering, Brown University, Providence, Rhode Island, United States of America; 3 Harvard-MIT Health Sciences and Technology, Cambridge, Massashusetts, United States of America; University of Reading, United Kingdom

## Abstract

Decellularization and cellularization of organs have emerged as disruptive methods in tissue engineering and regenerative medicine. Porous hydrogel scaffolds have widespread applications in tissue engineering, regenerative medicine and drug discovery as viable tissue mimics. However, the existing hydrogel fabrication techniques suffer from limited control over pore interconnectivity, density and size, which leads to inefficient nutrient and oxygen transport to cells embedded in the scaffolds. Here, we demonstrated an innovative approach to develop a new platform for tissue engineered constructs using live bacteria as sacrificial porogens. *E.coli* were patterned and cultured in an interconnected three-dimensional (3D) hydrogel network. The growing bacteria created interconnected micropores and microchannels. Then, the scafold was decellularized, and bacteria were eliminated from the scaffold through lysing and washing steps. This 3D porous network method combined with bioprinting has the potential to be broadly applicable and compatible with tissue specific applications allowing seeding of stem cells and other cell types.

## Introduction

Porous materials are of scientific and technological interest and find broad applications in multiple areas such as storage, separation, catalytic technologies as well as emerging microelectronics and medicine [1], [2], [3], [4], [5]. A disruptive shift in regenerative medicine has been observed moving from the use of synthetic implants and grafts towards the increased application of porous scaffolds with cells and biomolecules [3], [6], [7]. Recently, decellularized scaffolds have brought a new direction to this field [8], [9], [10]. This paradigm demands scaffolds that merge temporary structural and mechanical function with mass transport to enable tissue regeneration, where the dynamic pore features (*e.g.*, size, distribution) play an important role. For example, the optimal pore size of porous hydrogels has been shown to be in the range of 100–400 µm for cell seeding and tissue engineering applications [11], [12], [13], [14] and <100 µm for other applications including wound healing (optimal size 20–120 µm [15]) and vascularization (5–15 µm) [16]. Large pores in the scaffold surface that are interconnected to the inner pores are needed for controlled cell seeding and uniform cell distribution.

**Figure 1:**
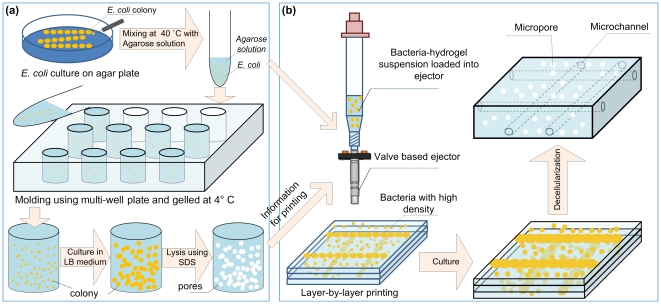
Illustration of the fabrication steps of microporous hydrogel scaffolds using living sacrificial porogens. (**a**) *E. coli* encapsulation in hydrogels as porogens and l**iv**e sacrificial pore formation. *E. coli* cultured on LB agar plate were collected and mixed with the agarose solution. After mixing, *E. coli* suspension was poured into a 12-well plate and solidifies. *E. coli* encapsulated in hydrogels were continuously cultured to allow formation of colonies. The living porogens were then lysed and the debris of *E. coli* and its DNA were removed by sequential washing with DPBS and DI water. (**b**) Formation of microfluidic channels. A line of *E. coli* / agarose mixture solution was printed onto Petri dish pre-coated with a layer of agarose. Then, another layer of agarose was used to cover the bacterial line. The hydrogels were gelled under rapid cooling (4°C) overnight.

Although leaching [17], [18], [19], gas foaming [20], [21], photolithography [22], polymer–polymer immiscibility [23], [24], freeze-drying [25] and emulsification [26], [27] methods have been utilized to introduce micropores into hydrogel scaffolds, a straightforward approach to prepare controllable porous hydrogels for broad biological applications has not been broadly achieved. Hydrophobic nanoparticles have been encapsulated in cell-laden hydrogels to enhance hydrogel permeability by loosing crosslinking density at the particle-hydrogel interface [28]. Recently, hydrogels (*e.g.*, PEG, alginate) have been used as soft porogens to fabricate continuous, open-pore geometry [29], [30], [31], [32], due to the deformation of the soft porogen material when packed. These gels have been used for tissue engineering of myocardium [33], cartilage [6] and brain tissue [34]. However, this approach is limited with inconsistent overall gel structure that varies from fibrous to a foam-like depending on the conditions used, partly due to the limited control over the spatial deposition of porogens. One existing challenge is that these methods cannot change the pore size within the same materials in a broad size range dynamically. Such a capability is needed to attain desired mechanical and physical properties of porous scaffold [3], and would benefit multiple organ systems. Freezing-thawing based methods have also been developed to fabricate supermacroporous interconnected-pore gels (*i.e.*, cryogels) [35], [36], [37], [38], [39]. The interconnected pores are formed through phase separation of gel precursor solution with ice-crystal formation via freezing, cross-linking and polymerization at sub-zero temperatures, and following ice-crystal thawing. However, the details fort each step during cryogel formation are still not clear which limits its wide applications [40]. Therefore, hydrogel scaffolds with programmable pore size and tunable pore geometry within a biological length scale (*i.e.*, mimicking the size of seeded cells and capillaries; tens to hundreds of micrometers) remain as an unmet need [32], [41], [42], [43]. To address this challenge, we introduced a living sacrificial porogen approach to fabricate three-dimensional (3D) hydrogel scaffolds embedded with interconnected micropores and microchannels (**Figure 1**).

## Materials and Methods

### Preparation of hydrogels and *E. coli*

Low temperature gelling agarose was used (Type VII-A,#A0701, Sigma Aldrich, St. Louis, MO) because of its availability from nature, biocompatibility [44], [45], adjustable mechanical properties [46], and diffusion properties [47], [48]. The gel was prepared under the same protocol as previously described [46]. Agarose hydrogels of different concentrations (weight/volume) were prepared by dissolving 1 g (1%) or 2 g (2%) agarose in 100 mL of deionized (DI) water and heating the mixture at ∼60°C in a microwave oven (Model M0902SST-1, Avanti, Miami, Florida) for 4 minutes.

*E. coli* (strain of JM103) were used in this study as living porogens. *E. coli* were cultured in Luria Broth (LB) EZMix™ medium (L7658, Sigma Aldrich, St. Louis, MO) and incubated overnight at 37°C at 225(RPM in a shaker incubator (SI4, Shel Lab, Cornelius, OR). The *E. coli* culture was then centrifuged and the supernatant was aspirated. The cell pellets were uniformly smeared in LB Agar plates (L1110, Teknova, San Diego, CA) in laminar hood to avoid contamination. The plated *E. coli* was cultured overnight in incubator at 37°C to form single *E. coli* colonies. *E. coli* colonies of size (∼2.5 mm in diameter) were scrapped off the LB Agar plate using the aseptic technique.

### Encapsulation of sacrificial bacteria in hydrogel

The bacterial colonies were collected and mixed with agarose solution and vortexed at 37°C to obtain a specified concentration of agarose-bacteria mixture (9.5×10^7^, 1.9×10^8^, 3.8×10^8^ CFUs/mL), **Figure 1**a. 2 mL of *E. coli* – agarose mixture solution was poured into each well of a 24-well plate (Cat. 3527, Corning Inc., Corning, NY). The mixture solution was gelled under rapid cooling in refrigerator (4°C) overnight, which has been shown to maintain cell viability with mamalian cells in our previous study [46], [49]. Bacteria encapsulating hydrogel samples of cylinderical shape of thickness 1.5 mm were prepared using a 10 mm punch (P1025, Acuderminc. USA). *E. coli* encapsulating agarose was cultured in LB medium and incubated at 37°C to enable colony formation.

Gram staining of *E. coli* colonies encapsulated in the hydrogel was performed according to the manufacturer's instructions (Procedure No. HT90, Sigma Aldrich, St. Louis, MO). The staining was carried out by immersing the hydrogel sample in crystal violet solution for 5 minutes, washing the sample with deionized water, and then repeating the same procedure with Safranin O solution (counter staining, Sigma Aldrich, St. Louis, MO). After staining, *E. coli* cells within the hydrogel were imaged with an inverted microscope (Nikon, TE2000).

### Patterning sacrificial porogens in 3D using cell printing

The strategy here is to use a cell printing system developed in our lab [50], [51], [52], [53] to deposit hydrogel encapsulated living sacrificial porogens at defined positions, **Figure 1b**. For this, *E. coli* was suspended in 0.5% agarose solution at a concentration of 8.0×10^8^ CFUs/ml. The petridishes were coated with 2% Agarose followed by 1% Agarose (0.5 mm in thickness) to give the mechanical stability by manual pipetting. The *E. coli* - agarose micture was pipetted into a 10 ml syring connected to a valve based ejector (G100–150300, TechElan, Mountainside, NJ). The printing system was sterilized with 70% ethanol and flushed with DI water before and after each ejection and constantly heated with a heating pad during ejection to avoid gelling of agarose. *E. coli* encapsulating agarose droplets were printed with 200 µs pulse width, 5 psi valve pressure and 10 Hz ejection frequency. To print a continuous line, the stage was programmed to move at a speed of 20 mm/s so that the neighboring droplets were in contact with each other. After gelling for 15 minutes at 37°C in incubator, the printed line was covered with 1% agarose and 2% agarose to provide stability. The sapmples were moved to a shaker incubator (SI4, Shel Lab, Cornelius, OR) for culture.

### Decellularization of cell-laden hydogels

To ensure complete lysis of *E. coli* in the agarose gel, the prepared hydrogel scaffold was immersed in 5% sodium dodecyl sulfate (SDS) for 12 hours, which has been shown to be able to diffuse into hydrogels and lyse the bacteria [54]. Next, the hydrogel scaffold was washed to remove *E. coli* debris with 1× DPBS (Cat. 14190, Invitrogen, Carlsbad, CA) for 2 hours and then with deionized water for 2 hours.

### Scanning electron microscopy (SEM) characterization

The agarose hydrogels were decellularized and washed with sterile PBS as described above. The control and porous agarose hydrogels were cut into cylindrical shapes with 8 mm diameter sterile biopsy punches and lyophilized (Labconco Corporation, Kansas City, MO) for 48 hours. Next, the lyophilized hydrogel sponges were placed in tightly closed containers for storage. For imaging the cross-sections, the lyophilized hydrogels were submerged into liquid nitrogen for 2 minutes. Next, hydrogels were freeze-fractured with sterile scalpel blades while submerged in liquid nitrogen, followed by air-drying in a low humidity hood for 30 minutes. The sectioned and dried hydrogels were mounted on 10 mm aluminum SEM stubs (Ted Pella Inc., Redding, CA) with double sided carbon tape (Ted Pella). The mounted samples were sputter coated (Cressington Scientific Instruments Ltd., Watford, England) with Platinum/Palladium at 40 mA for 90 seconds in a chamber purged with Argon gas. After sputter coating, the samples were imaged with field emission SEM (Ultra 55, Carl Zeiss MicroImaging, LLC, Thornwood, NY) under high vacuum mode with secondary electron detector.

### Bacterial live/dead staining

A bacterial cell Live/Dead BacLight™ kit (Cat. L7007, Invitrogen, Carlsbad, CA) was used to test the viability of *E. coli* encapsulated in hydrogels during culturing. The kit consisted of SYTO9 (10 µM in DI water), which stains the live bacteria cells with green fluorescence by penetrating intact membranes, and propidium iodide (PI, 110 µM in deionized water), which stains the dead cells with red fluorescence by entering compromised membranes. The samples were stained for 15 minutes using the live/dead kit and imaged using an inverted microscope (Nikon TE 2000).

### Characterization of colony density and size

The colony diameter and density were measured using the image processing software ImageJ (Rasband, W.S., ImageJ, U.S. National Institutes of Health, Bethesda, Maryland, USA, http://rsb.info.nih.gov/ij/, 1997–2009).

### Characterization of diffusion profile

After lysis and leaching of *E. coli*, the porosity of hydrogel scaffold was studied via the diffusion profile of a fluorescence dye, FITC Dextran (0.25 mM), which has a similar molecular weight of (20 kDa) to soluble growth factors associated with metabolism in the human body. The hydrogel samples (1.5 mm in thickness and 10 mm in diameter) were prepared using a razor blade. A 2 mm channel was punched in the center of gel for loading the fluorescence dye as a diffusion source. Diffusion of FITC Dextran into agarose samples was tested in a static condition to avoid the effect of non-pure diffusion (e.g., convection) induced by the surface roughness. Fluorescence images were taken under an inverted microscope (Nikon, TE2000) every 2 minutes. Constant volume of the dye in the source channel was maintained by refilling every 3 minutes. Fluorescent images were analyzed using the NIH ImageJ software to quantify spatial-temporal distribution of the diffusing dye in the gels.

### Characterization of mechanical properties

Samples with a cylinder shape of 1.5 mm in height were prepared using a 10 mm cylinder punch (Acu-Punch, Acuderm Inc., FL) and a razor blade. Both the height and diameter of each scaffold were measured using a digital micrometer (with accuracy of 0.01 mm) after putting the sample between glass slides in un-deformed state (before testing). Five sample dimension measurements were performed and the average value was used. The unconfined compression (i.e., without limiting lateral expansion) tests were performed to get the mechanical stiffness using an Instron™ material testing machine (Model 5542, Norwood, MA) with the Merlin™ software used to control the loading. The compressive moduli of lysed agarose gels were obtained from the linear regime of the stress-strain curves (10–15% strain). Each test was repeated three times with three samples and the results were reported as average ± standard deviation (STD).

### Polymerase chain reaction (PCR) test

To remove *E. coli* from hydrogels, the scaffold was treated with 5% SDS at room temperature overnight. PCR test was then performed to check if there is any DNA contamination from lysed *E. coli*. Briefly, hydrogel scaffold was excised with a clean blade, and the excised hydrogels were dissolved in buffer PB from MinElute Gel Extraction Kit (Qigen, Valencia, CA) at 55°C for 10 minutes. Dissolved hydrogel samples were then used to extract *E. coli* DNA using GenElute™ Bacterial Genomic DNA kit (Sigma-Aldrich, St. Louis, MO). The PCR reaction was performed targeting 16s RNA gene, as previously reported [55]. In a 50 µL PCR reaction, 25 µL of SYBR® Green PCR Master Mix, 2 µL of forward (5′-GAGGAAGGIGIGGAIGACGT-3′) and reverse primers (5′-AGICCCGIGAACGTATTCAC-3′) (both at a final concentration of 0.2 µM), 19 µL of H_2_O and 2 µL of DNA samples were added. The real-time PCR reaction was performed on 7300 Real-Time PCR System (Applied Biosystems, Carlsbad, CA) under the following conditions: 95°C for 10 minutes, and for 40 cycles of 95°C for 15 seconds and 60°C for 60 seconds.

### *In vitro* endotoxin test

Bacterial endotoxins that may remain in the scaffold after bacteria decellurization are the lipoolysaccharides (LPS), which is present in the cell membrane of the gram negative bacteria *E. coli*. To test the residual endotoxin, the limulus amebocyte lysate (LAL) test was performed with E-TOXATE (Sigma-Aldrich). Following the company protocol (http://www.sigmaaldrich.com/etc/medialib/docs/Sigma/Bulletin/et0300bul.Par.0001.File.tmp/et0300bul.pdf), all glassware was kept in the oven at 80°C for an hour and autoclaved for one hour at 121°C. The LAL test was performed in a sterile hood to avoid contamination. The agarose hydrogel samples were prepared under sterile conditions. Endotoxin standard solutions from the company were prepared by diluting the standard endotoxin stock solution with E-TOXATE water from the company. The lysate solution was mixed with the standard solutions at a ratio of 1∶1 (vol/vol) in a disposable culture tube. The mixture was then incubated for 60 minutes at 37°C. The tube was inverted slowly 180°C and the results were observed visually for the presence of a stable solid clot. A clotted incubation mixture is considered to be a positive result, while a result is negative, if an intact gel is not formed.

### Mammalian cell seeding

We used NIH-3T3 murine embryonic fibroblasts (CRL-1658, ATCC, Manassas, Virginia) to investigate the biocompability of fabricated porous hydrogel scaffolds. The cells were cultured in 3T3 medium consisting of 90% Dulbecco's Modified Eagle Media (DMEM, Gibco), 9% Fetal Bovine Serum (FBS, Gibco), and 1% penicilin-streptomycin (Sigma Aldrich St. Louis, Missouri) in an incubator (Model 3110, FormaScientific, Marietta, Ohio) at 37°C with 5% CO_2_. Once confluent, the cells were collected. After bacterial lysis, agarose scaffolds were washed five times in DPBS and twice in 3T3 cell media. The cylinder-shaped agarose scaffolds (10 mm in diameter and 1.5 mm in thickness) were put into 24-well plate (Corning Inc.). The cells (3T3) were seeded as a suspension (2×10^5^ cells/ml) to the hydrogels surface at a density of 4×10^4^ cells/cm^2^. After seeding for four hours, the unattached cells were washed away twice with DPBS. The samples were then submerged in 3T3 cell culture medium for subsequent culture. The cells were then visualized using an inverted microscope (Nikon TE 2000).

### Cell viability of seeded 3T3 cells

To check the viability of 3T3 cells seeded on porous agarose scaffolds, a fluorescent live/dead viability staining kit (Invitrogen, Carlsbad, CA) was used at day 1, 4, 7 and 14. The samples were washed once with DPBS and incubated in live/dead staining solution (0.5 µL calcein and 2.0 µL ethidium homodimer-1 (ETH) diluted in 1 mL DPBS) for 10 min (37°C, 5% CO_2_). The samples were washed with DPBS prior to imaging using an inverted microscope (Nikon, TE 2000). Live and dead cells were stained green and red, respectively. 3T3 cells grown on the hydrogels, which contained no *E. coli* in the hydrogel were used as controls.

### Statistical analysis

The experimental results were first tested for normal distribution with Anderson-Darling normality test. Colony diameter, colony density and area percentage (n = 10) were analyzed statistically with one way analysis of variance (ANOVA) with Tukey post-hoc comparisons for repeated measures. The compressive modulus values (n = 3) were tested with non-parametric Kruskal Wallis one way analysis of variance test and was found to be insignificant. Hence, pairwise comparisons were not performed. Statistical significance threshold was set at 0.05 for all tests (with p<0.05). Error bars in the figures, represent standard deviation. For mechanical measurement results, since the sample size was small (n = 3), non parametric Kruskal Wallis one way analysis of variance was performed and the statistical threshold values were found to be insignificant.

## Results and Discussion

**Figure 2:**
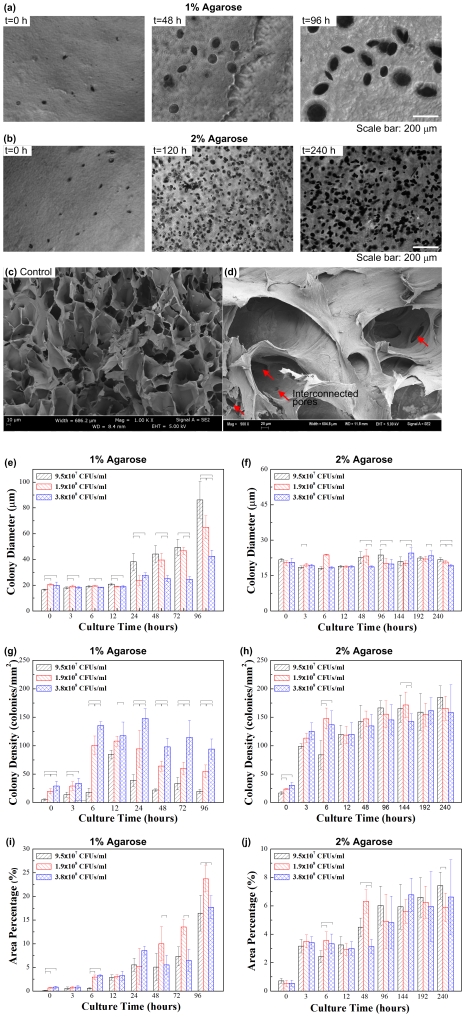
Characterization of living porogen growth in hydrogels and subsequent pore formation. Crystal violet staining of bacterial colonies in 1% (**a**) and 2% (**b**) hydrogels. SEM images of pores created using living porogens (**c**) as compared to controls (**d**). The colony size (**e–f**), density (**g–h**), and the pore area percentage (**i–j**) are presented over the culture time for 1% and 2% hydrogels at initial bacterial seeding concentrations of 9.5×10^7^, 1.9×10^8^ and 3.8×10^8^ CFUs/ml.

To investigate the growth characteristics of porogens encapsulated in hydrogels (1% and 2% agarose), we monitored colony size and density over time (0–96 hours for 1% agarose and 0–240 hours for 2% agarose), **Figure 2**. Temporal variations in these parameters were analyzed statistically by utilizing one way ANOVA with Tukey post-hoc comparisons with statistical significance set at 0.05 (p<0.05). We observed an increase in colony size (diameter) and colony density (number of colonies per mm^2^) in 1% agarose hydrogels and an increase in colony density in 2% agarose hydrogels at three initial porogen seeding densities (9.5×10^7^, 1.9×10^8^, 3.8×10^8^ CFUs/mL) (**Fig. 2**). In 1% agarose gel, colony diameter displayed a significant increase after 12 hours, followed by a secondary increase after 72 hours for seeding densities: 1.9×10^8^, 3.8×10^8^ CFUs/mL. However, for seeding density of 1.9×10^8^ CFUs/mL, colony diameter displayed a one-time constant increase after 24 hr (**Fig. 2a**). In the 2% agarose gel, the colony size (∼20 µm diameter) was similar to that in 1% agarose during the first 6 hours of culture (**Fig. 2b**). We observed that the colonies in 1% hydrogels were larger in diameter (up to 90 µm) and had a lower density at later stages of culture (t = 48, 96 hours) compared to 2% hydrogels. To investigate the microarchitecture of the porous hydrogels, we obtained SEM images of these gels (**Fig. 2c–d**). We observed enhanced porosity and interconnected pores (**Fig. 2d**) in the fabricated hydrogels using living porogens as compared to controls (**Fig. 2c**). In addition to the enhanced porosity, we also observed that the rest of the pore walls in **Figure 2d** show smoother surfaces as compared to **Figure 2c**. This may be due to the remodeling within bacteria-gel with the residing bacteria, where further studies on nano and microscale properties of this gel would be beneficial.

We observed that lower initial porogen concentrations resulted in a larger average colony size and hence larger pore size (**Fig. 2e–f**), potentially due to the competition among microorganisms for nutritional demand. At t = 96 hours for 1% agarose, the colony diameter was 86.3±14.3 µm for 9.5×10^7^ CFUs/mL, 65.0±8.9 µm for 1.9×10^8^ CFUs/mL, and 42.5±4.8 µm for 3.8×10^8^ CFUs/mL (**Fig. 2e**). However, we observed that there was no significant change of colony size and pore diameters in 2% hydrogels (in the range from 18.1 µm to 24.5 µm) with culture time and the initial bacterial concentration (p>0.05, **Fig. 2f**). This observation was potentially due to higher gel modulus in 2% agarose compared to 1% agarose that limits the growth and merging of adjacent colonies. The effect of gel modulus on bacterial growth was also reflected in the colony density (**Fig. 2g–h**). We observed that higher initial bacterial concentration resulted in a higher colony density in 1% agarose (**Fig. 2g**), while the colony density increased with culture time and reached to a maximum at t = 3 hours in 2% agarose (**Fig. 2h**). We observed no significant difference in colony density between gel groups using different initial bacterial concentrations, since the diffusion characteristics of the hydrogels were affected both by the pore size and density. The hydrogel porosity is determined by the volumes taken by the bacterial colonies in the hydrogels. To characterize this, we calculated the percentage of pores within the total volume using arial ratios. The results demonstrated that the area percentages taken by bacterial colonies increased over culture time, **Figure 2i–j**. Therefore, with the living sacrificial porogens, it was shown that control over pore size in hydrogels can be achieved by regulating initial seeding density, gel density, culture time or a combination of these parameters.

We also performed statistical analysis for colony diameter increase in 1% and 2% agarose (**Fig. 2e–f**). Two classes of comparison were made by varying the parameter. In one set, we fixed the colony size and compared pore diameter with culture time. In another set of study, the culture time was fixed and pore diameter was compared with the three different colony sizes (9.5×10^7^, 1.9×10^8^, 3.8×10^8^ CFUs/ml). 1 way ANOVA was chosen since the values were found to be normal using Anderson Darling's normality test. This indicated that culture time had a significant impact on the pore diameter.

For 1% agarose (**Fig. 2e**), in case of bacterial cell density of 9.5×10^7^ CFUs/ml, we observed that there was no statistical difference in colony diameter from 0 to 24 hours after which the diameter almost doubles. There was no increase in pore diameter between 24 hours and 48 hours. However, after 96 hours, the diameter was 85 µm which had a 4 fold increase. Interestingly, for the colony size of 1.9×10^8^ CFUs/ml, we noticed that there is statistically no difference in diameter from 0 to 48 hours after which there is a two fold increase in diameter. In addition, there is a significant increase in diameter for 72 hours and 96 hours. Therefore, statistical analysis clearly demonstrates that the culture time has an impact on colony diameter only after 24 hours for the colony size of 3.8×10^8^ CFUs/ml after which it didn't show significant change. At 96 hours, we observed a dramatic increase in colony diameter. Results of pair wise comparison show that at 0 hour, there was significant difference in pore diameter between 9.5×10^7^ and 1.9×10^8^ CFUs/ml and between 1.90×10^8^ and 3.8×10^8^ CFUs/ml. Same pattern was observed for culture time of 3, 12, 24 hours. On the contrar, for 6 hours of culture, a statistical difference was observed between 9.5×10^7^, 1.90×10^8^ and 3.8×10^8^ CFUs/ml. For culture time of 48 hours, significant difference was seen between 1.90×10^8^ and 3.8×10^8^ CFUs/ml, 9.5×10^7^ and 3.8×10^8^ CFUs/ml. In addition, culture time of 72 hours showed the same trend. All the three groups were found significant at a culture time of 96 hours.

An interesting observation was noted for 2% agarose gel (**Fig. 2f**) where in the case of 9.5×10^7^ CFUs/ml. There was no significant effect of culture time on the gel until 48 hours. The diameter increased dramatically at 96 hours and became constant beyond that time point. However, for colony size of 1.9×10^8^ CFUs/ml, there was not a significant change in pore diameter until 48 hours after which there was no signifcant change. We also observed an increase at 192 hours, beyond which it does not change significantly. In case of 3.8×10^8^ CFUs/ml, we observed a significant increase in pore diameter only after 144 hours. On the other hand, the pore diameter decreased significantly at 240 hours, due to the competition for nutrition and space. Results of pair wise comparison show that at 0, 6, 12 hours culture time there was no significant difference in pore diameter between different bacterial concentrations. On the contrary for 6 hours of culture, a statistical difference was observed between 9.5×10^7^, 1.9×10^8^, 3.8×10^8^ CFUs/ml. For culture time of 48 hours, significant difference was observed between 1.9×10^8^ and 3.8×10^8^ CFUs/ml, 9.5×10^7^ and 3.8×10^8^ CFUs/ml. Culture time of 96 hours showed significant comparison between 9.5×10^7^ and 1.9×10^8^ CFUs/ml, 9.5×10^7^ and 3.8×10^8^ CFUs/ml. For culture time of 144 hours and 192 hours, significant difference was observed between 9.5×10^7^ and 1.9×10^8^ CFUs/ml, and between 1.9×10^8^ and 3.8×10^8^ CFUs/ml respectively. All the three groups were found significant at a culture time of 240 hours.

**Figure 3:**
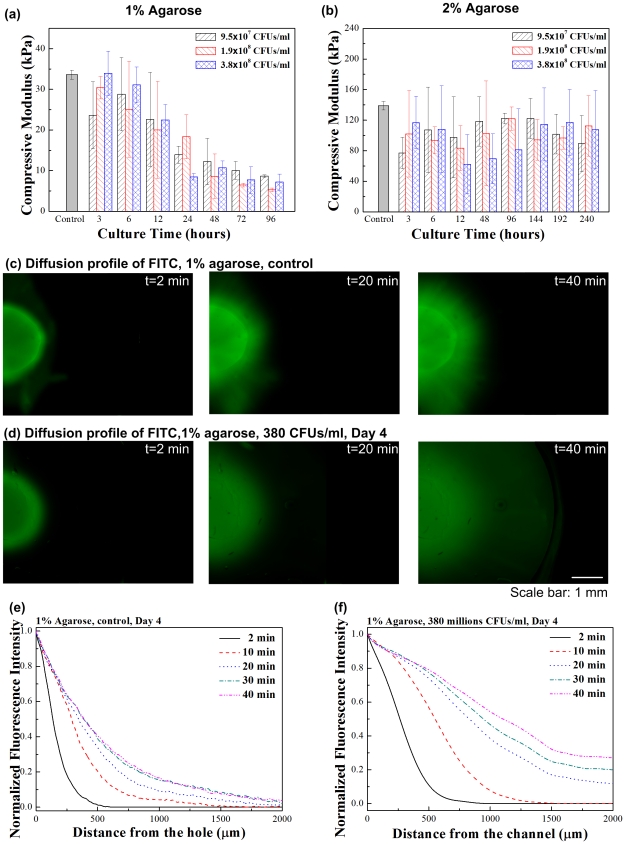
The mechanical stiffness (a–b) and perfusion properties (c–f) of porous hydrogels. Compressive moduli were inversely correlated with the culture time for 1% hydrogel (**a**), whereas there was no significant change in stiffness for 2% hydrogel (**b**). Fluorescence images of FITC-dextran (0.25 mM, 20 kDa) diffusion in the 1% porous agarose hydrogels (**c**) and controls (**d**). The diffusion profiles of FITC-dextran as a function of distance from the source channel in porous (**e**) and non-porous (**f**) hydrogels. (n = 3).

To investigate the effect of initial porogen concentration and culture time on the mechanical stiffness of the fabricated porous agarose gels, we performed compressive testing using an Instron mechanical tester. 2% agarose gels displayed significantly higher compressive modulus (139.0±5.8 kPa) (**Fig. 3b**) compared to 1% non-porous agarose (33.6±1.1 kPa) (**Fig. 3a**). Bacterial culture time has an effect on compressive stiffness of 1% agarose gel, with a decreasing compressive modulus over culture time (p<0.05). For 1% agarose, the moduli were 30.4±2.8 kPa, 18.3±5.4 kPa, 5.3±0.5 kPa for culture time of 3, 24 and 96 hours, respectively (**Fig. 3a**). On the other hand, the compressive modulus of 2% agarose was not significantly affected from bacteria culture duration (p>0.05). For 2% agarose, the moduli were 102.1±56.6 kPa, 102.8±68.8 kPa, 96.7±14.7 kPa for culture durations of 3, 48 and 192 hours, respectively (**Fig. 3b**). These results agreed well with the bacterial colony growth in **Figure 2e–j** and indicated that mechanical properties of the porous hydrogels can be controlled by culture duration. Hydrogels are viscoelastic materials. In this study, we measured compressive modulus to investigate the bacterial colony growth within the hydrogels, as hydrogel modulus/elasticity has been widely used as a parameter for characterizing cell mechanical microenvironment [56].

To evaluate the created micropores for the diffusion of soluble molecules within agarose gels, we analyzed the diffusion profiles using a fluorescent dye FITC-Dextran. Unprocessed agarose gels were used as controls. We observed that FITC-Dextran diffused faster in porous hydrogels compared to controls (**Fig. 3c–d**). The corresponding spatial-temporal diffusion profiles were also characterized as a function of distance from the channel boundary, where the FITC-Dextran dye solution was loaded (**Fig. 3e–f**). The improved diffusion of FITC-dextran through the gels with enhanced pores is expected since the path length through the gel is shortened due to the existence of pores. Although this does not measure porosity and does not necessarily suggest interconnectivity, diffusion behavior reflects the total percentage pore volume.

**Figure 4:**
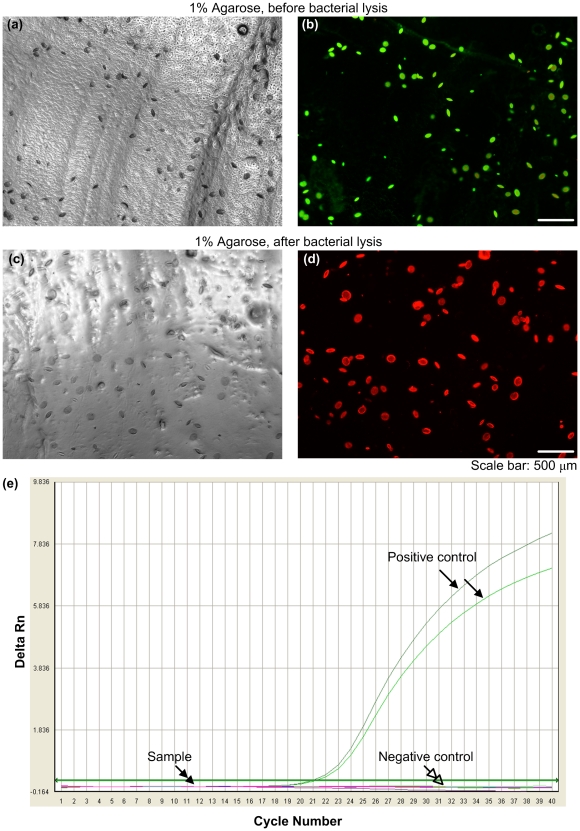
Lysis of *E. coli* in 1% hydrogel. Hydrogels containing *E. coli* were imaged under bright-field before (**a–b**) and after lysis (**c–d**). The hydrogels were also stained using bacterial cell Live/Dead BacLight™ kit and then imaged under green and red fluorescence filters before and after lysis (**c–d**). Live *E. coli* cells are shown in green and dead *E. coli* cells are shown in red. (**e**) PCR results. PCR test was performed to check the DNA contamination from lysed *E. coli* targeting 16s RNA gene. There was no detectable 16s RNA gene in the porous hydrogel.

We achieved bacterial decellularization by perfusing the hydrogels with SDS, which has been successfully used to decellularize whole organs from xenogenic sources (*e.g.*, heart [8], liver [9]) and effectively remove cellular constituents compared to other detergents (*e.g.*, polyethylene glycol, Triton-X100, enzyme-based protocols) [8]. To assess the efficiency of bacterial lysis during the decellularization in this study, we tested the viability of *E. coli* in agarose hydrogels, **Figure 4**. We observed that bacteria cultured in hydrogels were live before lysis process (**Fig. 4a–b**), and all were dead after lysis (**Fig. 4c–d**). Further, we checked for the presence of 16s RNA gene of *E. coli* using realtime PCR. The results showed no detectable 16s RNA gene in the porous hydrogel, indicating that *E. coli* DNA was removed after lysis and washing steps (**Fig. 4e**). Although the remnant bacterial proteins and lipids may not be detectable in the wash solution from the hydrogels, they may still exist at trace levels within the hydrogels. Although these may not be directly toxic, they might have the potential to cause retained biomolecules or generalized pyrogen activity. Complete removal and suppression of these potential remainders are critical for a biocompatible scaffold matrix. Therefore, we performed LAL endotoxins test to investigate the presence of residual bacterial endotoxins (LPS present in the cell membrane of the gram negative bacteria *E. coli*) in the scaffold. We observed no gel coagulation which indicated no detectable bacterial endotoxins in the porous agarose scaffolds after the decellularization process.

**Figure 5:**
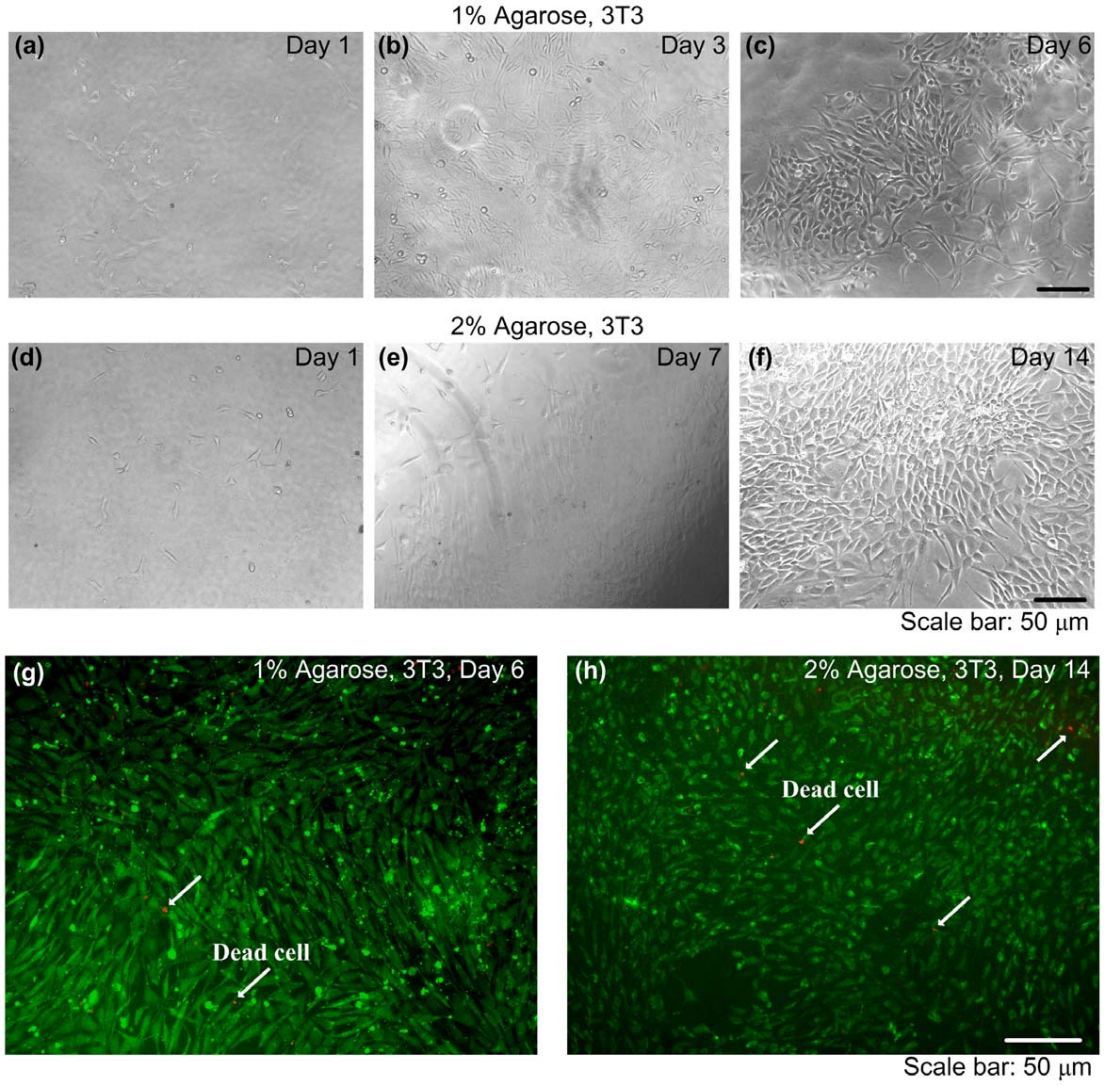
Biocompatibility of fabricated porous hydrogels. After lysis of porogens, the porous scaffolds were washed with DPBS and cell medium. 3T3 were then seeded on the scaffold and cultured. 3T3 cells proliferated and were confluent on day 6 for 1% hydrogel (**a–c**) and on day 14 for 2% hydrogel (**d–f**). Cells were alive after confluence (**g–h**).

To assess the mammalian cell growth and viability on the fabricated porous scaffold, we seeded 3T3 cells on the hydrogel surface and monitored them in long-term cultures (**Fig. 5**). 3T3 cells proliferated and became confluent on day 6 on 1% agarose gel (**Fig. 5a–c**) and day 14 on 2% agarose (**Fig. 5d–f**). These results were similar to the controls without bacterial exposure. Cells stay alive for 2 weeks indicating the biocompatibility of these scaffolds (**Fig. 5g–h**). The test showed that the decellurized hydrogel was biocompatible and allowed cellular growth when the cells are seeded on top of the gel. The duration of the cultures and the cellular affinity of the biomaterial used will influence the cell migration into the gel.

**Figure 6:**
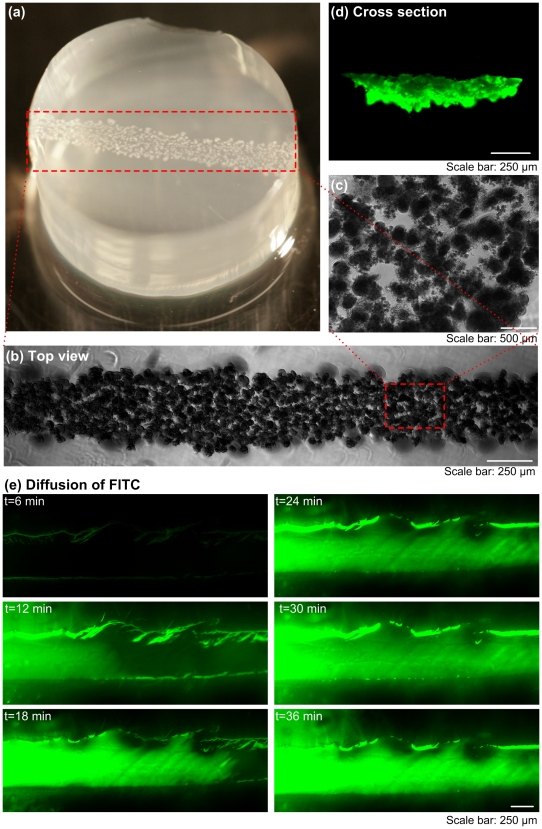
Microchannel formation by spatially bioprinting the sacrificial living porogens using a cell bioprinter. (**a**) *E. coli* (800,000 CFU/mL) were mixed with 0.5% agarose at 40°C. Agarose-bacteria mixture was printed on a 1% agarose pre-coated petri dish and covered with another 1% agarose layer on top. (**b**) Top view of bacterial colony chain in 0.5% agarose. (**c**) Merged bacterial colonies. (**d**) Cross-section of a formed microchannel in the hydrogel. (**e**) Diffusion enhanced in bioprinted microfluidic hydrogels at areas with high porous density.

To create localized and interconnected high porosity regions and demonstrate the spatial control over the density and location of the pores in hydrogels, we directly patterned *E. coli* encapsulated in droplets at high concentrations (8.0×10^8^ CFUs/mL) using a cell printing approach [52], [57] by encapsulating cells in droplets [51] (**Fig. 1b**, **Fig. 6**). We observed that the printed bacteria formed colonies that merged over a period of culture time (**Fig. 6b–c**). After 4 days of culture, we observed a continuous line of bacterial colonies (**Fig. 6a**). 3D channel geometry was further confirmed by the cross sectional images (**Fig. 6d**), and diffusion experiment (**Fig. 6e**).

The sacrificial porogen method uses living microorganisms porogens that were seeded with spatial and density control into hydrogels via a bioprinter, followed by a culture period. After sacrificing and removing the microorganisms (*i.e.*, decellularization), micropores and microchannels were generated within the hydrogels. Decellularization has been used to engineer complex whole organs such as heart [8], liver [9] and lung [10] with preserved microarchitecture of the original tissue such as native microvasculature. Using the same decellularization process, the living porogen approach can dynamically and spatially control the pore size and density as a function of culture time and seeding density of microorganisms. The porogens formed interconnected pores when distributed in hydrogels and formed microchannels when localized at high densities. In addition, this methodology is cost effective, as it does not require specific equipment and is compatible with standard hydrogel fabrication techniques.

Cell printing is a novel technology capable of patterning multiple cell types in 3D scaffolding materials (e.g., hydrogels) *in vitro* at **high throughput**. In our laboratory, we have developed a cell printing system that can pattern various cell types (smooth muscle, stem cells, cancer cells, fibroblasts) with high viability (>90%) and maintained functionality [52], [53], [58], [59]. In this study, we used this cell printing system to precisely position sacrificial *E. coli* according to the predetermined design. We fabricated simple straight channels. We anticipate that this approach can be applied to fabricate complex 3D architectures with a layer by layer printing and cell encapsulation strategy [60], [61], [62], [63] such that the features (e.g., size, density, distribution) of embedded pores and channels can be regulated by only changing the printing cell density.

Microorganisms have been widely applied in biomedical arena. For instance, *Escherichia coli (E. coli)* have been investigated for applications in biotechnology and in material science, such as biorobotics [64], gene identification [65], and biosensors [66], [67]. However, even though bacterial cultures have been traditionally performed on agar plates (*i.e.*, a typical hydrogel), the incorporation of hydrogels with living microorganisms to create 3D porous scaffolds has not been systematically studied. Therefore, incorporation of microorganisms as temporary and sacrificial porogens in hydrogels can be a facile approach to achieve controlled porosity, pore interconnectivity and adjustable mechanical properties of scaffolds giving a dynamic control over the scaffold design properties. Further, the United States Food and Drug Administration has approved the use of several microorganism in food products such as *Lactobacillus*, *Bifidobacterium*, *Streptococcus Thermophilus*, *Salmonella*, yeast, and bacteriophage [68], and these microorganisms can be also used as living porogens, although we only utilized *E. coli* in this study as a model.

We demonstrated here a living sacrificial porogen method to fabricate porous hydrogels with dynamically controllable embedded micropores and microchannels through controlling seeding density and culture time in hydrogels that were decellularized. The presented approach is a first in terms of controlling the pore distribution, size, density within a single biomaterial combined with a biopatterning method, leading to temporal and density dependent parameters to control mechanical and diffusion characteristics of hydrogels. The developed approach could potentially be a broadly applicable biotechnology tool for fabricating porous hydrogels with potential impact on multiple fields including regenerative medicine, drug and cell delivery therapies and pharmaceutical research.
